# Human serum‐derived exosomes modulate macrophage inflammation to promote VCAM1‐mediated angiogenesis and bone regeneration

**DOI:** 10.1111/jcmm.17727

**Published:** 2023-03-25

**Authors:** Xi Xiang, Janak Lal Pathak, Wenbin Wu, Jianwen Li, Wenyan Huang, Qiuyu Wu, Mengyu Xin, Yuejun Wu, Yuhang Huang, Linhu Ge, Sujuan Zeng

**Affiliations:** ^1^ Department of Pediatric Dentistry, Affiliated Stomatology Hospital of Guangzhou Medical University, Guangdong Engineering Research Center of Oral Restoration and Reconstruction Guangzhou Key Laboratory of Basic and Applied Research of Oral Regenerative Medicine Guangzhou China

**Keywords:** angiogenesis, bone regeneration, human serum exosomes, macrophage inflammation regulation, VCAM1

## Abstract

During exogenous bone‐graft‐mediated bone defect repair, macrophage inflammation dictates angiogenesis and bone regeneration. Exosomes from different human cells have shown macrophage immunomodulation‐mediated bone regeneration potential. However, the effect of human serum‐derived exosomes (serum‐Exo) on macrophage immunomodulation‐mediated angiogenesis during bone defect repair has not been investigated yet. In this study, we explored the effects of serum‐Exo on macrophage inflammation regulation‐mediated angiogenesis during bone defect repair and preliminarily elucidated the mechanism. Healthy serum‐Exo was isolated by ultracentrifugation. The effect of serum‐Exo on LPS‐induced M1 macrophage inflammation was analysed in vitro. The conditioned medium of serum‐Exo‐treated LPS‐induced M1 macrophage (serum‐Exo‐treated M1 macrophage‐CM) was used to culture human umbilical vein endothelial cells (HUVEC), and the effect on angiogenesis was analysed by western blot, qRT‐PCR, etc. mRNA‐sequencing of HUVECs was performed to identify deferentially expressed genes. Finally, the rat mandibular defect model was established and treated with Bio‐Oss and Bio‐Oss + Exo. The effect of the Bio‐Oss + Exo combination on mandibular bone regeneration was observed by micro‐computed tomography (micro‐CT), haematoxylin and eosin (HE) staining, Masson staining, and immunohistochemical staining. Serum‐Exo promoted the proliferation of RAW264.7 macrophages and reduced the expression of M1‐related genes such as IL‐6, IL‐1β, iNOS, and CD86. Serum‐Exo‐treated M1 macrophage‐CM induced the proliferation, migration, and angiogenic differentiation of HUVEC, as well as the expression of H‐type blood vessel markers CD31 and endomucin (EMCN), compared with M1 macrophage‐CM. Moreover, higher expression of vascular endothelial adhesion factor 1 (VCAM1) in HUVEC cultured with serum‐Exo‐treated M1 macrophage‐CM compared with M1 macrophages‐CM. Inhibition of VCAM1 signalling abrogated the pro‐angiogenic effect of serum‐Exo‐treated M1 macrophage‐CM on HUVEC. Local administration of serum‐Exo during mandibular bone defect repair reduced the number of M1 macrophages and promoted angiogenesis and osteogenesis. Collectively, our results demonstrate the macrophage inflammation regulation‐mediated pro‐angiogenic potential of serum‐Exo during bone defect repair possibly via upregulation of VCAM1 signalling in HUVEC.

## 1 INTRODUCTION

Exogenous bone grafts such as 3D‐printed scaffolds or xenogenic bone grafts (e.g. Bio‐Oss) are frequently used for bone defect repair in the clinic.^1^, ^2^ Although these bone grafts are designed to exert minimum immunogenicity, the graft recipient's immune system treats these grafts as foreign bodies and exerts inflammatory responses.^3^ Macrophages are the key immune cells that actively take part in osteoimmunology during bone graft‐mediated bone defect repair.^3^ Based on the immunogenicity of graft material and sensitivity of immune systems, macrophages polarize to either pro‐inflammatory M1 phenotype or anti‐inflammatory M2 phenotype.^4^ The majority of exogenous bone grafts induce M1‐macrophage polarisation in vivo.^5^ Although M1 macrophages are required during a very early stage of bone repair, prolongation of M1 macrophage polarisation during the early, middle and late stages of bone repair creates an inflammatory bone micro‐environment that hinders bone defect repair.^3^ Inflammatory mediators released from M1 macrophages inhibit migration, proliferation and differentiation of precursor cells including mesenchymal stem cells (MSCs) and endothelial cells during bone defect repair.^6^, ^7^ Therefore, regulation of bone‐graft‐mediated macrophage immunomodulation during bone defect repair is currently at the centre of attention.

Angiogenesis promotes bone regeneration during bone defect repair via osteogenesis‐angiogenesis coupling.^8^ Type‐H vessels characterized by CD31^hi^ and EMCN^hi^ expressing endothelial cells are mainly responsible for osteogenesis and angiogenesis coupling during bone defect repair.^9^ Reports from the literature showed an inhibitory effect of M1 macrophages in angiogenesis during bone defect repair.^10^ Therefore, mitigation of M1 macrophages could be beneficial to promote not only osteogenesis but also angiogenesis during bone defect repair. Various anti‐inflammatory exogenous growth factors including IL‐10 and IL‐4 are used to inhibit M1 macrophage polarisation during bone‐graft‐mediated bone regeneration.^11^ However, these exogenous growth factors are relatively expensive and also pose the risk of local and systemic adverse effects.^12^ Therefore, novel cost‐effective approaches to mitigate M1 macrophages during bone defect repair are highly demanded in clinics.

Exosomes are nano‐scale vesicles secreted by cells, with a diameter of about 100 nm.^13^ Exosomes encapsulate nucleic acids, proteins, lipids, amino acids and metabolites, and other components, and participate in various physiological and pathological processes.^13^ Exosomes from different human cells including MSCs and M2 macrophages had shown inflammation regulation and immunomodulation potential in vitro and in vivo studies.^14^, ^15^ However, exosomes from allogenic cells exert immunogenicity, and the isolation of enough amount of exosomes from autologous cells source is time‐consuming, highly expensive, and requires a highly sophisticated GMP level laboratory setup. Moreover, the use of exogenous growth factors and cell culture conditions to expand cells in vitro might exert adverse effects in vivo. Human serum contains a large number of exosomes and can be quickly and easily isolated in a simple laboratory setup that provides the opportunity of using autologous serum‐Exo to treat various diseases. Therefore, it is wise to investigate the macrophage inflammation mitigation potential of serum‐Exo during bone graft‐mediated bone defect repair.

This study aimed to (i) isolate and characterize serum‐Exo from healthy individuals; (ii) investigate the effect of serum‐Exo on macrophage inflammation regulation; (iii) analyse the effect of serum‐Exo on macrophage inflammation‐mediated angiogenesis during bone defect repair. Serum‐Exo inhibited LPS‐induced macrophage inflammation to promote angiogenesis in Bio‐Oss‐grafted bone defect repair via upregulation of VCAM1 signalling in endothelial cells, suggesting the possible application of autologous serum‐Exo to mitigate exogenous bone graft‐induced‐immunogenicity‐mediated macrophage inflammation.

## 2 MATERIALS AND METHODS

### 2.1 Cell culture

RAW264.7 murine macrophage cell lines and HUVEC primary cells were purchased from Procell Life Science &Technology Co., Ltd (Procell CL‐0190) and the National Collection of Authenticated Cell Cultures, respectively. Cells were cultured in Dulbecco's modified Eagle medium (DMEM) high glucose medium supplemented with 10% foetal bovine serum (FBS) (BioInd), 100 U/mL of penicillin, and 100 μg/mL of streptomycin or in an endothelial cell special medium (ECM) at 37°C in a humidified 5% CO_2_/95% air atmosphere.

### 2.2 Isolation and characterisation of serum‐Exo

A total of 40 healthy volunteers that is, 20 males and 20 females were recruited to collect the blood sample. Inclusion criteria for the volunteers were as follows: (i) healthy individuals with age 20–30 years, (ii) no history of smoking, (iii) blood platelets count >10^5^/μL cells. Persons with a history of infectious diseases, systemic diseases, and haematological disorders and under medication such as Asprin in the last 3 months were not included in this study. Five milliliters of blood was collected from each volunteer in a vacutainer tube without anticoagulants and placed vertically for 30 min at room temperature. The clotted blood was centrifuged at 1000 rpm for 10 min and serum was collected. This study was approved by the Medical Ethics Committee of the Affiliated Stomatology Hospital of Guangzhou Medical University (LYCJ2021026). Informed consent was obtained from all the volunteers.

Serum samples were centrifuged at 20,000 *g* for 45 min. The supernatant was transferred to an ultracentrifuge tube (Beckman). Samples were then ultracentrifuged at 110,000 *g* at 4°C for 60 min.^16^ The obtained serum‐Exo was dissolved in phosphate‐buffered solution (PBS) for subsequent experiments, and their total protein concentration of them was quantified by the BCA Protein Assay Kit (Beyotime). Four concentrations (25, 50, 100, and 200 μg/mL) of serum‐Exo were tested for macrophage inflammation inhibition study.

Serum‐Exo size distribution was measured by the Nanoparticles Tracking Analysis (NTA) and Zeta Potential Distribution Analyzers (Paricle Metrix‐PMX). Serum‐Exo morphology was visualized under high‐resolution transmission electron microscopy (TEM, Hitachi HT7700). Western blot (WB) analysis detected the expression of serum‐Exo markers CD9, CD63, CD81, and TSG‐101. Finally, according to the product instructions, 1 μL of PKH26 (Sigma‐Aldrich) diluted in 250 μL of Diluent C was incubated with the isolated serum‐Exo at room temperature. Five minutes later, 1% BSA was used to halt the staining. The mixture was centrifuged and washed two times with DMEM by centrifuging at 100,000 *g* for 2 h. The PKH26‐labelled serum‐Exo was resuspended in 1 mL of culture medium and added to RAW264.7. After 24 h of incubation, the cells were fixed using 4% paraformaldehyde for 15 min and subsequently stained by 4′,6‐diamidino‐2‐phenylindole (DAPI). PKH26‐labelled serum‐Exo internalisation in macrophages was visualized under a confocal microscope (Leica).

### 2.3 Flow cytometry

After the RAW264.7 cells were cultured under the 6‐well plates after stimulation by lipopolysaccharide (LPS; Sigma) for 24 h. The cell suspension was incubated with the blocking antibody CD16 (Abcam) and the anti‐mouse CD86 (PE; Abcam) and sorted in a flow cytometry machine as soon as possible.

### 2.4 Conditioned medium preparation

To harvest the conditioned medium (CM), RAW264.7 were incubated with 100 ng/mL LPS for 24 h to induce macrophage inflammation (M1 macrophages) and then substituted for a fresh culture medium with or without containing 50 μg/mL serum‐Exo for 24 h. Then, the medium was replaced with a serum‐free endothelial cell culture medium (ECM). At 6 h, the cell culture supernatant was collected and centrifuged at 1000 rpm for 5 min to remove detached cells and cellular debris. At this time, processed supernatant was mixed with ECM serum‐free medium at a ratio of 1:1 and configured into the CM with or without serum‐Exo pre‐treatment, which was M1 macrophage‐CM and serum‐Exo‐treated M1 macrophage‐CM, respectively. CM was stored at −80°C for future experiments.

### 2.5 Cells proliferation, migration, and tube formation

Cell Counting Kit‐8 (CCK8, Dojindo) was used to evaluate RAW264.7 cells proliferation using serum‐Exo (50 μg/mL). In 96‐well plates, RAW264.7 or HUVEC (3000 cells/well) were cultured. Cells were treated in the dark for 2 h with a 10% (v/v) CCK8 solution. A microplate reader (SPECTRO star Nano) was used to determine the absorbance at 450 nm.

In the wound healing experiment, HUVEC was inoculated on a 24‐well plate to scrape the joined cells with a 200 μL pipette tip after fusion and take pictures. Images were taken under a light microscope after 6 or 12 h of incubation in M1 macrophage‐CM or serum Exo‐treated M1 macrophage‐CM and the wound healing rate was determined.

In the transwell migration assay, a different conditioned medium containing 10% foetal bovine serum (600 μL) is added to the lower chamber of the 24‐well transwell insert (8 μm pore size, Corning Costar), and the HUVEC suspension containing 5% foetal bovine serum (200 μL) was seeded into the upper chamber at a density of 1 × 10^5^ cells/well. Migrated cells were analysed accordingly.

In the Matrigel tube formation assay, Matrigel and ECM for free‐serum in a 1:1 ratio (10 μL/well, BD, USA) were added to precooled ibiTreat plates and polymerized at 37°C for 30 min to form a thin gel layer. HUVEC (1 × 10^5^ cells/well) were suspended in a conditioned medium supplemented with 5% FBS and seeded onto ibiTreat plates containing the mixture above. After being incubated for 2.5 h, the capillary‐like structures were observed under a light microscope, and pictures of 3–5 visual fields/well were taken. The total length of the tubular structure was analysed by using the Angiogenesis Analyser plug‐in for ImageJ software (NIH).

### 2.6 Aortic ring assay

Thawed matrigel (100 μL) was transferred into the central 10 wells of a 48‐well plate on ice. Subsequently, the plate is transferred to 37°C and placed for 30 min to allow it to solidify. The rat aorta was placed on the laid matrigel and then added 100 μL matrigel to the rat aorta, allow to solidify at 37°C for 30 min. M1 macrophage‐CM or serum‐Exo‐treated M1 macrophage‐CM (200 μL) were added to each test well. After a further 6–8 days, all plates were fixed with 4% paraformaldehyde, which was washed extensively with water. Explants and their outgrowths were photographed under an optical microscope, allowing the entire well to be imaged.

### 2.7 Quantitative real‐time PCR

Total cellular RNA was isolated from RAW264.7 cells or HUVEC with SteadyPure Quick RNA Extraction Kit (RightGene) and then reverse‐transcribed into cDNA with PrimeScript® RT reagent kit (Takara). Afterward, RT‐qPCR was performed with SYBR Premix Ex TaqTM (Tsingke). Analysis was performed on RT‐qPCR System (Bio‐Rad). The housekeeping gene GAPDH was used for normalisation. The primers used in this study are shown in Table 1.

**TABLE 1:** Primers used for RT‐qPCR analysis.

Gene	Acc. No	Primer sequence (5′‐3′)	Product length (bp)
Mu *GAPDH*	NM_001289726.1	Forward: AAGAAGGTGGTGAAGCAGG	111
		Reverse: GAAGGTGGAAGAGTGGGAGT	
Hu *GAPDH*	NM_001127501.4	Forward: GGACCATTCCCACGTCTTCAC	137
		Reverse: CCTTGTAGCCAGGCCCATTG	
Mu *iNOS*	NM_001313922.1	Forward: ACTCAGCCAAGCCCTCA	105
		Reverse: CTCTGCCTATCCGTCTCGT	
Mu *IL‐1β*	NM_008361.4	Forward: ACAGGCTCCGAGATGAACAA	212
		Reverse: GGGTGTGCCGTCTTTCATT	
Mu *IL‐6*	NM_001314054.1	Forward: TCCATCCAGTTGCCTTCTTG	106
		Reverse: GGTCTGTTGGGAGTGGTATC	
Hu *VEGF*	NM_001025366.3	Forward: GGAGGCAGAGAAAAGAGAAAGTGT	175
		Reverse: TAAGAGAGCAAGAGAGAGCAAAAGA	
Hu *VEGFR‐2*	NM_002253.4	Forward: ACGGACAGTGGTATGGTTCTTGCC	145
		Reverse: GGTAGCCGCTTGTCTGGTTTGAG	
Hu *vWF*	NM_000552.5	Forward: AGCCCATTTGCTGAGCCTTG	115
		Reverse: CCTGGCACCATGCATTTCTG	
Hu *b‐FGF*	NM_002006.6	Forward: AGTCTTCGCCAGGTCATTGAGATC	160
		Reverse: CGTCCTGAGTATTCGGCAACAG	
Hu *EMCN*	NM_001159694.2	Forward: CAGCAACCAGCCGGTCTT	113
		Reverse: TGCCTTCCAGCACATTGG	
Hu *CD31*	NM_000442.5	Forward: CACAGCAATTCCTCAGGCTA	596
		Reverse: TTCAGCCTTCAGCATGGTAG	
Hu *VCAM1*	NM_080682.3	Forward: CAGTAAGGCAGGCTGTAAAAGA	137
		Reverse: TGGAGCTGGTAGACCCTCG	

### 2.8 Western Blot

The protein sample from cells or serum‐Exo (10 μg) was separated and transferred onto a 0.2‐μm PVDF membrane (Millipore), followed by blocking with QuickBlock™ Blocking Buffer (Beyotime) for 30 min. The primary antibodies include anti‐GAPDH (Abcam), anti‐CD9 (Abcam), anti‐CD81 (Abcam), anti‐CD63 (Abcam), anti‐TSG101 (Abcam), anti‐iNOS (Abcam), anti‐VEGF (Abcam), anti‐CD31 (Abcam), anti‐EMCN (Proteintech), anti‐VCAM1 (Proteintech) were then diluted according to the manual and incubated with the membrane at 4°C overnight. The membrane was then treated with a secondary antibody (Thermo Fisher Scientific) for 1 h at room temperature. The density analysis of bands shown on the ECL Western Blotting Substrate was performed using Image J software.

### 2.9 mRNA sequencing

Sequencing was performed by Nanjing Paisennuo Gene Technology Co., Ltd. After quality control of the original data, the high‐quality sequencing data were compared with the designated reference genome. The expression values were calculated by the StringTie tool, and the tDESeq algorithm was applied to filter the differentially expressed genes. Gene Set Enrichment Analysis (GSEA) and Kyoto Encyclopaedia of Genes and Genomes (KEGG) analyses were performed to reveal the involved pathways. Finally, the sequencing results were verified by RT‐qPCR and WB.

### 2.10 VCAM1 inhibition assay

Zaurategrast (CDP323) (MedChemExpress), an antagonist of the vascular cell adhesion molecule 1 (VCAM1) binding to alpha4‐integrins (other adhesion molecules), was used to assess the participation of VCAM1 in the serum‐Exo‐M1 macrophage‐CM‐mediated effects on HUVEC. Serum‐Exo‐treated M1 macrophage‐CM was pretreated with 5 μM CDP323 for 24 h. WB and RT‐qPCR were used to evaluate the angiogenic differentiation of HUVEC.

### 2.11 Animal experiment design

Bio‐Oss (Geistlich) were used as serum‐Exo carriers in our study. In total, 50 μg of serum‐Exo combined with 5 mg of Bio‐Oss were used for each defect. For the immobilisation of serum‐Exo, 50 μg/μL serum‐Exo was incubated with 5 mg of Bio‐Oss at 4°C overnight to enable complete loading of the scaffolds with serum‐Exo. The animal experiments involved in this project were approved by the Laboratory Animal Ethics Committee of Guangdong Huawei Testing Co., Ltd. (approval number: 20210903). A total of 10 male SD rats (8 weeks old) were accommodated in a specific pathogen‐free (SPF) environment and a bone defect of approximately 6 × 2 × 0.5 mm^3^ was created at the buccal of the anterior mandible.^17^ The rats were separated into two groups at random: (i) a group treated with Bio‐Oss (Bio‐Oss, *n* = 5); and (ii) a group treated with Bio‐Oss loaded with serum‐Exo (Bio‐Oss + serum‐Exo, *n* = 5). Bio‐Gide (Geistlich) was used to cover the defect in all rats in case the implants were removed. At 6 weeks following the surgery, the mandibles with the defects were obtained and fixed with 4% paraformaldehyde for 24 h. Subsequently, bone regeneration was assessed by Microcomputed tomography (micro‐CT), the defect healing and bone formation was observed by Haematoxylin & Eosin (HE) and Masson's staining, The amount of CD31, VCAM1 and iNOS was evaluated by Immunohistochemistry staining.

### 2.12 Statistical analysis

All experiments were repeated three times and all results are expressed as the mean ± SD. Statistical analysis was performed with GraphPad Prism 5.0 (GraphPad Software). The *t*‐test was used for statistical analysis between two groups, and the one‐way anova was used for statistical analysis between three or more groups. The test level was *α* = 0.05. Differences were considered statistically significant at *p* < 0.05.

## 3 RESULTS

### 3.1 Isolation and characterisation of serum‐Exo

**FIGURE 1:**
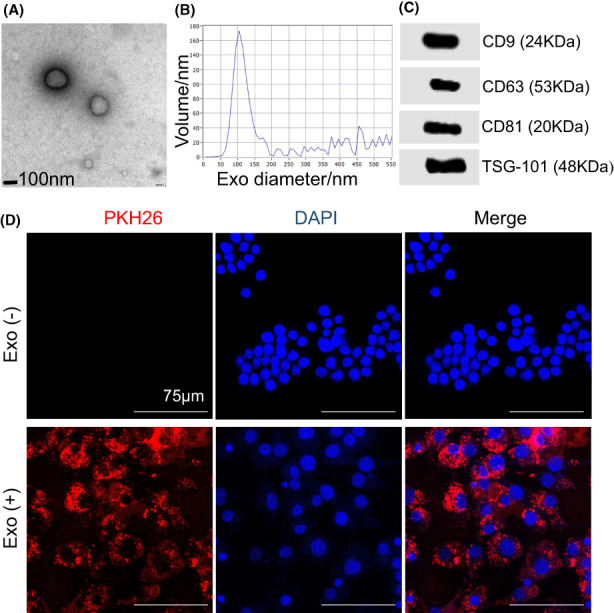
Characterisation of serum‐Exo and cellular internalisation in RAW264.7 cells. (A) Representative TEM images of serum‐Exo. (B) Particle size distribution of serum‐Exo measured by NanoSight analysis. (C) Western blot analysis of the serum‐Exosome surface markers. (D) Internalisation of serum‐Exo by RAW264.7 cells after 24 h of treatment.

Serum‐Exo was isolated from human serum by ultrafiltration centrifugation combined with ultracentrifugation. TEM analysis showed that serum‐Exo isolated from human serum bore a cup‐shaped morphology (Figure 1A). Size and concentration detection of serum‐Exo by NTA showed that the serum‐Exo had a narrow size distribution, with a mean particle diameter of 101.49 nm with a range of 50–150 nm (Figure 1B). Serum‐Exo markers CD9, CD63, CD81 and TSG101 were abundantly expressed in serum‐Exo (Figure 1C). The protein concentration of isolated serum‐Exo stock was 7.0 μg/μL.

### 3.2 Serum‐Exo was uptaken by the RAW264.7 cells

To study the uptake of isolated serum‐Exo, we treated serum‐Exo with PKH26, a fluorescent dye with long aliphatic tails that are incorporated into the lipid membrane of serum‐Exo.^18^ RAW264.7 cells were incubated with PKH26‐labelled serum‐Exo for 24 h. We observed the presence of PKH26‐positive granules in the cytoplasm of RAW264.7 cells by confocal laser microscopy, suggesting that RAW264.7 cells uptake the serum‐Exo (Figure 1D).

### 3.3 Serum‐Exo promoted RAW264.7 proliferation

**FIGURE 2:**
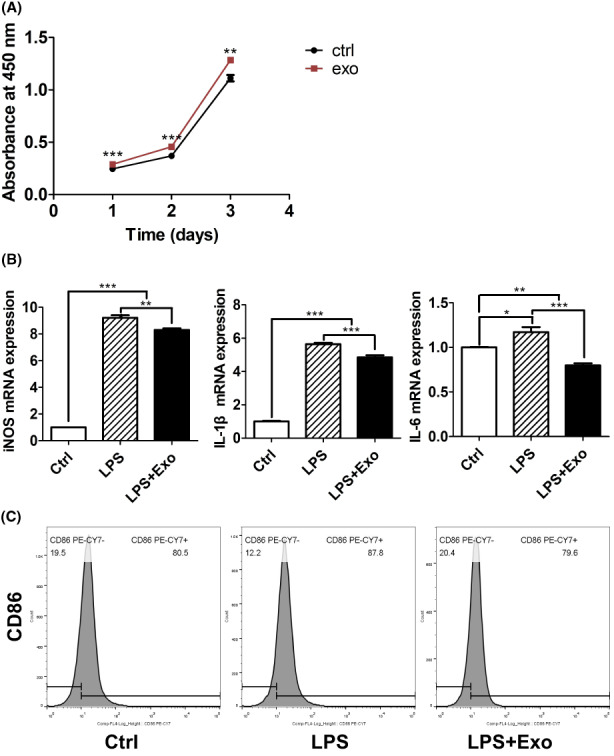
Effects of serum‐Exo on RAW264.7 macrophage viability and immunomodulation. (A) Cell viability analysed by CCK8 assay (*n* = 5). (B) RT‐qPCR analysis for M1 macrophage markers (*n* = 3). (C) Flow cytometry analysis of M1 macrophage marker CD86. Significant difference between the groups, *p < 0.05, **p < 0.01, and ***p < 0.001.

We selected 50 μg/mL as the optimal anti‐inflammatory concentration of serum‐Exo by RT‐qPCR analysis and used it in subsequent experiments (Figure S1A). CCK‐8 assays revealed that serum‐Exo significantly promoted the proliferation of macrophages cultured for day 1, 2 and 3 (Figure 2A).

### 3.4 Serum‐Exo mitigated LPS‐induced macrophage inflammation

LPS treatment induced the mRNA expression of inflammatory markers IL‐1β, IL‐6 and iNOS by 5.6‐, 1.2‐ and 9.2‐fold, respectively (Figure 2B). Serum‐Exo mitigated the LPS‐induced mRNA expression of IL‐1β, IL‐6 and iNOS by 1.2‐, 1.5‐ and 1.1‐fold, respectively. A similar effect of serum‐Exo was observed in the expression of inflammatory macrophage marker CD86 in FACs analysis (Figure 2C).

### 3.5 Serum‐Exo‐treated M1 macrophage‐CM promoted HUVEC proliferation and migration

**FIGURE 3:**
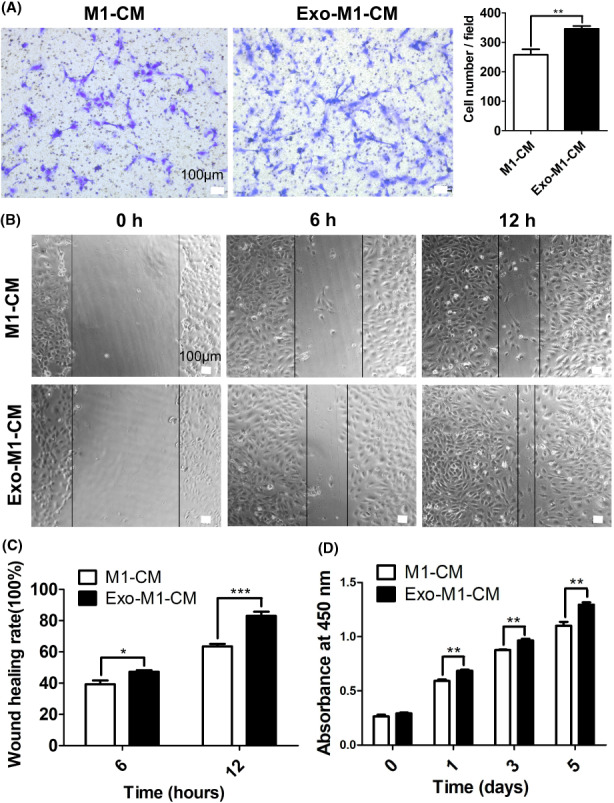
Serum‐Exosome‐treated M1 macrophage‐CM promotes HUVEC migration and proliferation. (A) Representative microscopic images and quantification of migrated cells in the transwell cell migration assay (*n* = 5). (B) Representative microscopic images of the wound‐healing assay. (C) Quantification of wound healing rate (*n* = 4). (D) HUVEC cell viability analysed by CCK8 assay (*n* = 4). Significant difference between the groups, *p < 0.05, **p < 0.01, ***p < 0.001.

Serum‐Exo‐treated M1 macrophage‐CM robustly promoted the migration of HUVEC compared with the M1 macrophage‐CM as indicated by the results of the transwell assay and wound healing scratch assay (Figure 3A–C). Moreover, HUVEC cultured with serum‐Exo‐treated M1 macrophage‐CM showed a higher proliferation compared with M1 macrophage‐CM‐treated HUVEC (Figure 3D). These results showed an anabolic effect of serum‐Exo‐treated M1 macrophage‐CM on the proliferation and migration of HUVEC compared to M1 macrophage‐CM.

### 3.6 Serum‐Exo‐treated M1 macrophage‐CM promoted angiogenic differentiation of HUVEC

**FIGURE 4:**
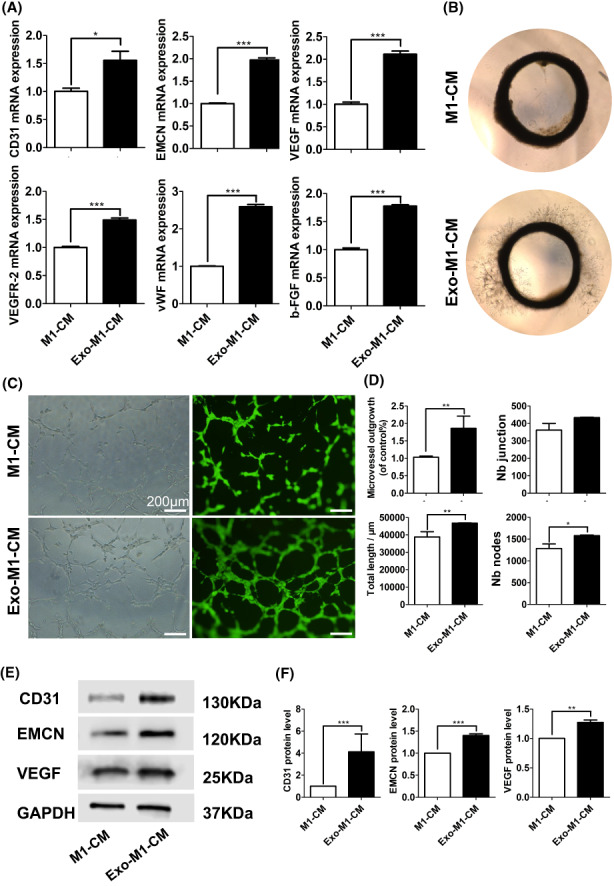
Serum‐Exosome‐treated M1 macrophage‐CM promotes angiogenic differentiation of HUVEC. (A) RT‐qPCR analysis of angiogenic differentiation markers (*n* = 3). (B) Representative microscopic images of SD rat arterial ring sprouting assay (*n* = 3). (C) Representative microscopic images of Matrigel tube formation assay. (D) Quantification of the newly formed tube in matrigel assay (*n* = 3). Representative Western blot images (E), and quantification (F) of angiogenic differentiation markers (*n* = 3). Significant difference between the groups, *p < 0.05, **p < 0.01, ***p < 0.001.

Serum‐Exo did not directly affect the angiogenic differentiation of HUVEC cells (Figure S1B). HUVEC cultured with serum‐Exo‐treated M1 macrophage‐CM showed a higher mRNA expression of angiogenic markers CD31 (1.6‐fold), EMCN (2.0‐fold), VEGF (2.1‐fold), VEGFR2 (1.5‐fold), vWF (2.6‐fold) and b‐FGF (2.6‐fold) compared with the M1 macrophage‐CM (Figure 4A). In addition, the aortic ring assay illustrated that the number of microvessel outgrowth around the aortic ring in the serum‐Exo‐treated M1 macrophage‐CM group was 1.9‐fold higher than that in the M1 macrophage‐CM group (Figure 4B,D). To further investigate the effect of serum‐Exo‐treated M1 macrophage‐CM on HUVEC angiogenesis in vitro, a Matrigel tube formation assay was performed. Images of the Matrigel assay showed more complete and round tubes in the serum‐Exo‐treated M1 macrophage‐CM group compared with the M1 macrophage‐CM group. The total tube length and the number of nodes were significantly increased in the serum‐Exo‐treated M1 macrophage‐CM group compared with the M1 macrophage‐CM (Figure 4C,D). The protein level expressions of VEGF, CD31 and EMCN were 1.3‐, 4.1‐ and 1.4‐fold higher, respectively in the serum‐Exo‐treated M1 macrophage‐CM group were compared with the M1 macrophage‐CM group (Figure 4E,F). These results indicate that serum‐Exo‐modulated macrophage inflammation promotes angiogenesis.

### 3.7 Serum‐Exo‐treated M1 macrophage‐CM promoted VCAM1‐mediated angiogenic differentiation of HUVEC

**FIGURE 5:**
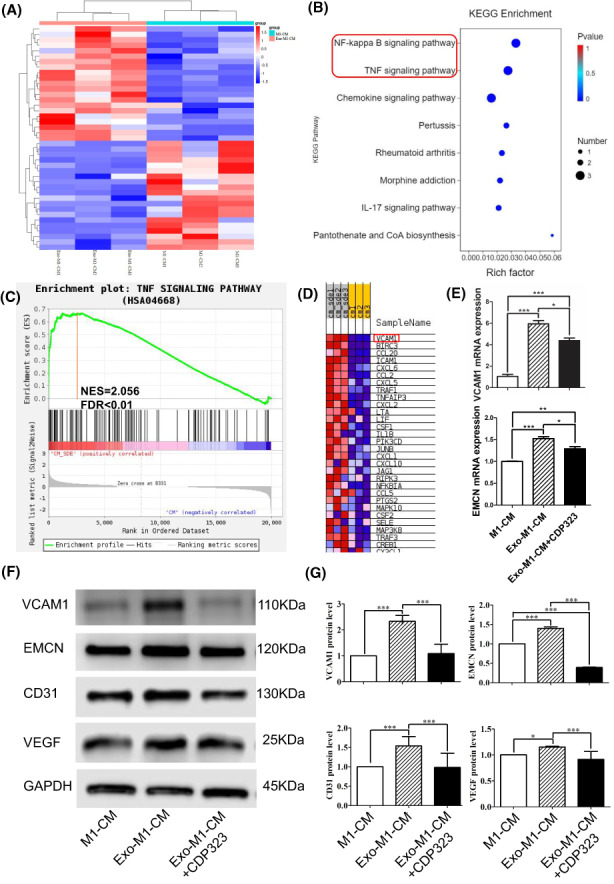
Serum‐Exosome‐treated M1 macrophage‐CM promotes angiogenic differentiation of HUVEC via VCAM1. (A) Heatmap of differentially expressed genes in HUVEC. (B) KEGG pathway enrichment analysis of differentially expressed genes. (C) Gene Set Enrichment Analysis of DEGs of TNF signalling pathway in HUVEC. (D) Gene set heatmap of TNF signalling pathway. (E) RT‐qPCR analysis of VCAM1 and EMCN expression in HUVEC (*n* = 3). Representative Western blot images (F), and quantification (G) of angiogenic differentiation markers (*n* = 3). CPD323: VCAM1 inhibitors. Significant difference between the groups, *p < 0.05, **p < 0.01, and ***p < 0.001.

To explore the differential gene expression of M1 macrophage‐CM and serum‐Exo‐treated M1 macrophage‐CM‐treated HUVEC during angiogenic differentiation transcriptomic sequencing was performed. The results showed that compared with the M1 macrophage‐CM group, a total of 44 genes were differentially up‐regulated, and 34 genes were differentially down‐regulated in the serum‐Exo‐treated M1 macrophage‐CM group compared to the M1 macrophage‐CM group (Figure S2). Among these the differentially expressed genes were involved in NF‐ĸB, TNF, rheumatoid arthritis, and chemokine signalling pathways as well as many other biological processes related to angiogenesis (Figure 5A,B). GSEA analysis showed that the highest scores of ENS were NF‐ĸB and TNF pathways, and VCAM1 was both enriched in these two pathways (Figure 5C,D). Inhibition of VCAM1 by CDP323 (VCAM1 inhibitor) inhibited the serum‐Exo‐treated M1 macrophage‐CM induced mRNA expression of VCAM1 and EMCN in HUVEC cells (Figure 5E). Similarly, inhibition of VCAM1 inhibited serum‐Exo‐treated M1 macrophage‐CM induced protein level expression of VCAM1, EMCN, CD31 and VEGF (Figure 5F,G). These outcomes indicated that VCAM1 is involved in serum‐Exo‐treated M1 macrophage‐CM mediated angiogenic differentiation of HUVEC.

### 3.8 Serum‐Exo promoted mandibular bone defect repair

**FIGURE 6:**
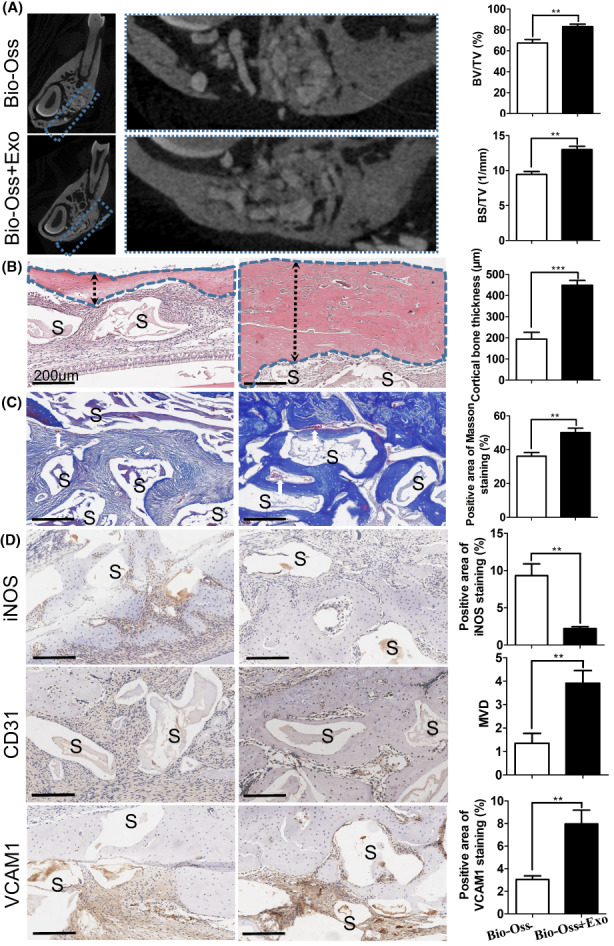
Human serum‐Exosome promotes osteogenesis and angiogenesis in rat mandibular bone defects. (A) Representative micro‐CT sectional view of the healing of mandibular bone defect in rats and quantitative analysis of newly formed bone parameters (*n* = 5). (B) Representative microscopic images of Haematoxylin & Eosin stained tissue sections and quantification of newly cortical bone thickness (*n* = 5). (C) Representative microscopic images of Masson Trichrome stained tissue sections and quantification of Masson stained newly formed bone (*n* = 5). (D) Representative microscopic images of immunohistochemistry of tissue sections and quantification of iNOS, CD31 and VCAM1 expression (*n* = 5). S: Bio‐Oss scaffold; white arrow: newly formed blood vessel. Significant difference between the groups, **p < 0.01 and ***p < 0.001.

Serum‐Exo was carried by Bio‐Oss in vivo to examine its therapeutic effects. In total, 10 SD rats with mandibular defects were split into two groups (Bio‐Oss and Bio‐Oss + serum‐Exo; *n* = 5). After 6 weeks, the mandibles with defects were harvested and scanned using micro‐CT. Figure 6A shows a 2D image of the bone defect area surrounded by blue boxes in each group. Compared to defects implanted with only Bio‐Oss, defects implanted with Bio‐Oss + serum‐Exo healed better. There was a marked increase in new bone formation in the Bio‐Oss + serum‐Exo‐treated group when compared to the Bio‐Oss group, particularly in terms of new bone thickness by 2.3‐fold (Figure 6B). Compared to Bio‐Oss, the Bio‐Oss + serum‐Exo‐treated group improved BV/TV and BS/TV ratio by 1.2, and 1.4‐fold, respectively, according to 3D reconstruction analysis (Figure 6A). According to Haematoxylin & Eosin staining, Bio‐Oss + serum‐Exo showed greater bone regeneration and thicker cortical bones than Bio‐Oss alone, and the latter group has more infiltrating inflammatory cells (Figure 6B). In the BioOss+serum‐Exo group, Masson staining showed more collagen deposition and blood vessels punctate with collagen (Figure 6C). Moreover, compared with the pure Bio‐Oss group, iNOS expression was downregulated by 4.2‐fold in the defect area of the Bio‐Oss + serum‐Exo group. Angiogenesis‐related proteins CD31 and VCAM1 displayed the opposite trend from iNOS expression (Figure 6D). These results indicate that the serum‐Exo promotes bone regeneration, downregulates macrophage inflammation and upregulates angiogenesis during critical‐sized bone defect repair.

## 4 DISCUSSION

**FIGURE 7:**
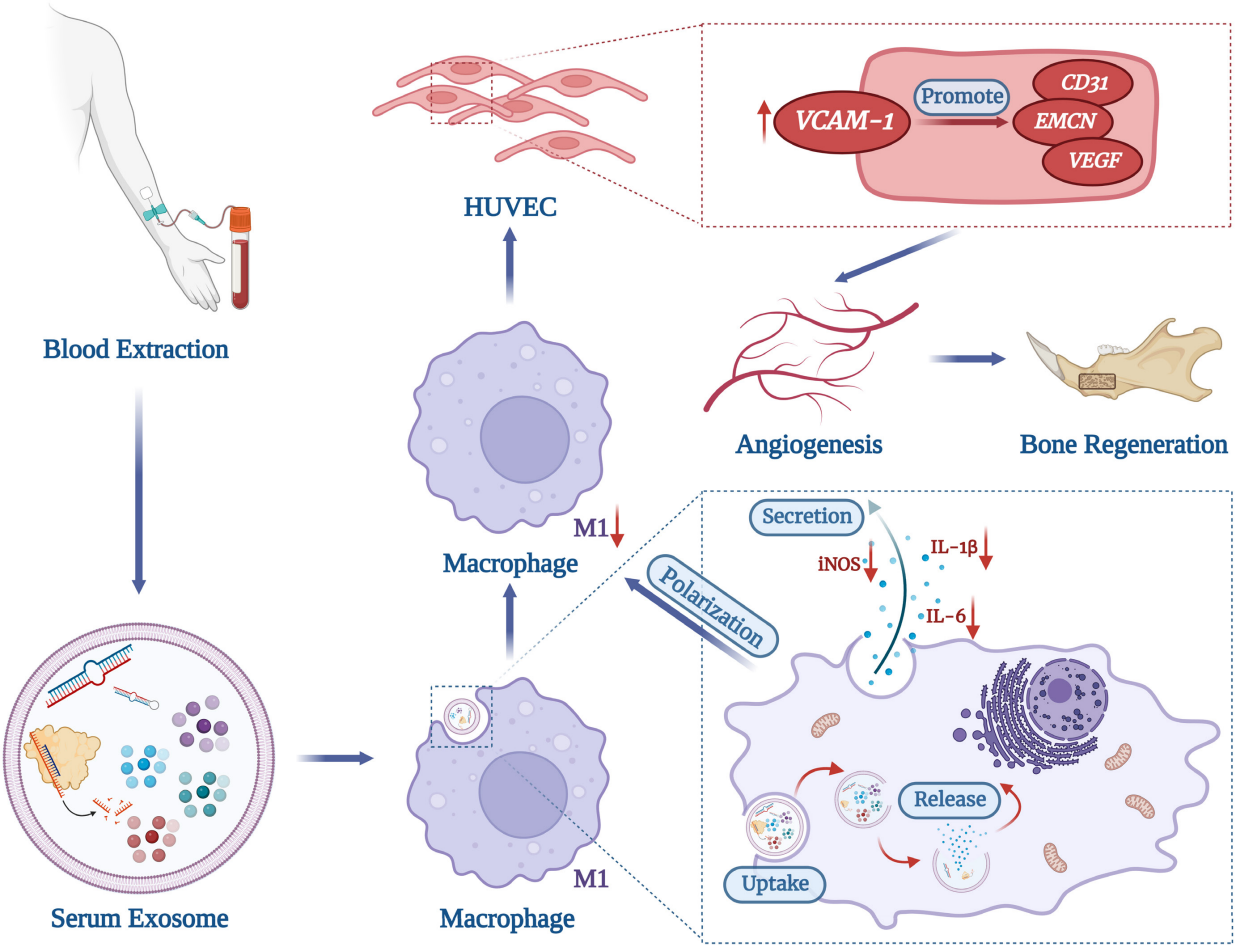
Scheme indicating the role of serum‐Exo on macrophage inflammation‐mitigation‐mediated angiogenesis during bone defect repair.

Serum‐Exo has shown tissue regenerative potential via regulating cells' functions.^19^ Serum‐Exo has been reported as a diagnostic/prognostic and therapeutic agent for the treatment of various diseases including cardiovascular diseases and diabetic wounds.^19^, ^20^ During bone‐graft material‐based bone defect repair, graft materials induce an inflammatory response of macrophages hindering angiogenesis and osteogenesis.^3^ Angiogenesis plays a crucial role in bone regeneration via osteogenesis‐angiogenesis coupling.^8^ However, the macrophage inflammation regulation‐mediated effect of serum‐Exo on angiogenesis during bone defect repair has not been investigated yet. Serum‐Exo inhibited macrophage inflammation both in vitro and in vivo. Serum‐Exo‐mediated inhibition of macrophage inflammation further induced angiogenesis both in vitro and during bone defect repair possibly via upregulation of VCAM1 in HUVEC (Figure 7). These results indicate the bone regeneration‐inducing potential of serum‐Exo via macrophage inflammation mitigation‐mediated angiogenesis.

Shreds of literature have reported the serum‐Exo isolation and characterisation procedures in various previous literature.^21^, ^22^ In this study, the size of serum‐Exo isolated by healthy donors by ultracentrifugation method ranged from 50 to 150 nm with a mean diameter of 101.49 nm. Serum‐Exo robustly expressed exosome‐specific markers CD9, CD63, CD81 and TSG‐101. These results are according to results from the previous literature.^23^ Serum‐Exo has been reported to regulate inflammation. Lu et al. showed that serum‐Exo from tumour patients promotes macrophage inflammation.^24^ While another study showed that rat‐derived serum‐Exo inhibits macrophage inflammation.^25^ This study found that serum‐Exo from healthy donors was efficiently internalized by macrophages. Moreover, serum‐Exo treatment alleviated LPS‐induced macrophage inflammation in vitro. Although serum‐Exo from a diseased individual exerts inflammatory effects or other catabolic effects,^24^ the anti‐inflammatory effect of serum‐Exo from a healthy individual can be utilized to treat various diseases and tissue regeneration applications. It is worth noting that serum‐Exo carries various components such as RNAs, proteins and enzymes which might have cellular activities in a concentration‐dependent manner. The macrophage inflammation inhibitory effect of 50 μg/mL but not by the 100 and 200 μg/mL of exosomes might be related to the concentrations of RNAs and proteins carried by different amounts of serum‐Exo. However, the component of serum‐Exo responsible for macrophage inflammation should be further investigated.

Newly formed blood vessels play an important role in bone regeneration via osteogenesis‐angiogenesis coupling.^8^ Newly formed type‐H vessels characterized by CD31^hi^ and EMCN^hi^ are mainly responsible for osteogenesis‐angiogenesis coupling during bone regeneration.^9^ However, effective angiogenesis during bone regeneration is still a challenging task in clinical practice. Numerous studies in regenerative medicine have shown that inflammation inhibits angiogenesis during tissue regeneration.^10^ The proliferation and migration of HUVEC are key processes required for angiogenesis.^26^ We found that serum‐Exo‐treated M1 macrophage‐CM promoted the migration and proliferation of HUVEC compared with the M1 macrophage‐CM. Moreover, serum‐Exo‐treated M1 macrophage‐CM promoted the expression of angiogenic differentiation markers in endothelial cells including type‐H vessel markers CD31 and EMCN compared with M1 macrophage‐CM. Serum‐Exo‐treated M1 macrophage‐CM also promoted angiogenesis both in vitro and in vivo compared with the M1 macrophage‐CM. Serum‐Exo mir‐340‐5p has been reported to promote angiogenesis in brain microvascular endothelial cells during oxygen–glucose deprivation. However, serum‐Exo did not directly promote the angiogenic differentiation of endothelial cells cultured with normal oxygen and glucose levels.^27^ Our results indicate the proangiogenic effect of serum‐Exo via inhibition of macrophage inflammation.

VCAM1 is an important pro‐angiogenic factor.^28^ Retinoblastoma cell derived‐exosomes induce VCAM1 expression in endothelial cells to promote angiogenesis.^29^ Transcriptome analysis, RT‐qPCR, and Western blot analysis confirmed a higher expression of VCAM1 in endothelial cells cultured with serum‐Exo‐treated M1 macrophage‐CM compared with M1 macrophage‐CM. Inhibition of VCAM1 abrogated the serum‐Exo‐treated M1 macrophage‐CM‐induced expression of angiogenic markers and type‐H vessel markers in endothelial cells. Our results confirm the role of VCAM1 in serum‐Exo‐treated M1 macrophage‐CM mediated angiogenesis.

Bone graft‐mediated bone regeneration is a complex process that involves the activity of osteogenic and angiogenic precursor cells as well as immune cells such as macrophages.^30^, ^31^ MSCs‐derived exosomes had shown bone regenerative potential via regulating the survival and activity of these cells.^32^, ^33^ In this study, local application of serum‐Exo promoted bone graft‐based rat mandibular bone defect repair. Enhanced VCAM1 expression and angiogenesis and alleviated macrophage inflammation were observed during bone defect repair in serum‐Exo‐treated rats. Results from the bone defect repair study were in accordance with the results from in vitro studies on serum‐Exo‐mitigated macrophage inflammation‐mediated angiogenesis. Our results indicate that serum‐Exo could be used to mitigate bone‐graft‐induced macrophage inflammation that further promotes angiogenesis during bone defect repair.

We extensively analysed the role of serum‐Exo on macrophage inflammation mitigation‐induced angiogenesis during bone defect repair using in vitro and in vivo studies. Moreover, compared with the direct effect of serum‐Exo on HUVEC, the application of serum‐Exo‐treated M1 macrophage‐CM has better angiogenic potential (Figure S1B). However, the key factor present in the serum‐Exo that mitigates macrophage inflammation should be further investigated. Moreover, the mechanism of serum‐Exo‐treated M1 macrophage‐CM‐induced VCAM1 in endothelial cells should also be explored.

Despite tremendous progress in deciphering the mysteries of exosomes over the past few decades, challenges in efficient exosome isolation remain unresolved.^34^ Although ultracentrifugation has been the ‘gold standard’ for exosome separation due to its high processing capacity, high levels of protein aggregate, and lipoprotein contamination in exosome samples prepared through this method greatly compromises their quantification and functional analysis.^35^ The development of standardized methods for exosome isolation for clinical application is an urgent task. Different storage temperatures and storage times affect the stability, size distribution, and number of particles of exosomes, as well as the cellular uptake and biodistribution of exosomes.^36^ In 2018, Chonlada et al. used trehalose as a cryoprotectant to freeze‐dry melanoma exosomes, which can be stored stably for 4 weeks at 25°C.^37^ However, whether lyophilisation affects exosome function and stability is still unknown. Therefore, universally accepted approaches for exosome isolation and storage are mandatory to translate exosome‐related research to clinical application.

## 5 CONCLUSION

Serum‐Exo inhibits macrophage inflammation to promote angiogenesis via the upregulation of VCAM1 in HUVEC. Serum‐Exo promotes bone regeneration and angiogenesis and alleviates macrophage inflammation during scaffold‐based bone defect repair. These results suggest the possible application of autologous serum‐Exo during scaffold‐based critical‐sized bone defect repair.

## ETHICS APPROVAL AND CONSENT TO PARTICIPATE

The study is approved by the Medical Ethics Committee of the Affiliated Stomatology Hospital of Guangzhou Medical University, approval number: LYCJ2021026. The animal experiments involved in this project were approved by the Laboratory Animal Ethics Committee of Guangdong Huawei Testing Co., Ltd. (approval number: 20210903).

## AUTHOR CONTRIBUTIONS

**Xi Xiang:** Data curation (equal); writing – review and editing (lead). **Janak L. Pathak:** Funding acquisition (equal); writing – review and editing (equal). **Wenbin Wu:** Data curation (equal); formal analysis (equal); methodology (equal). **Jianwen Li:** Data curation (equal); methodology (equal); software (equal). **Wenyan Huang:** Methodology (equal); supervision (equal); writing – review and editing (equal). **Qiuyu Wu:** Software (equal). **Mengyu Xin:** Methodology (equal). **Yuejun Wu:** Resources (equal); software (equal). **Yuhang Huang:** Software (equal). **Linhu Ge:** Project administration (equal); visualization (equal); writing – review and editing (equal). **Su juan Zeng:** Funding acquisition (equal); project administration (equal); validation (lead); writing – review and editing (equal).

## CONFLICT OF INTEREST STATEMENT

The author reports no conflicts of interest in this work.

## Supporting information


FigureS1‐S2

## Data Availability

The part of raw data generated or analyzed during this study is included in this published article and its supplementary information files. The raw data presented in tables are available from the corresponding author upon reasonable request.
