# Supplementary material for: Human serum‐derived exosomes modulate macrophage inflammation to promote VCAM1‐mediated angiogenesis and bone regeneration
Source: J Cell Mol Med. 2023 Mar 25;27(8):1131–43. doi: 10.1111/jcmm.17727 (PMC10098299; doi:10.1111/jcmm.17727)
Supplement: Supplementary file 1 — FigureS1‐S2 [file JCMM-27-1131-s001.docx]

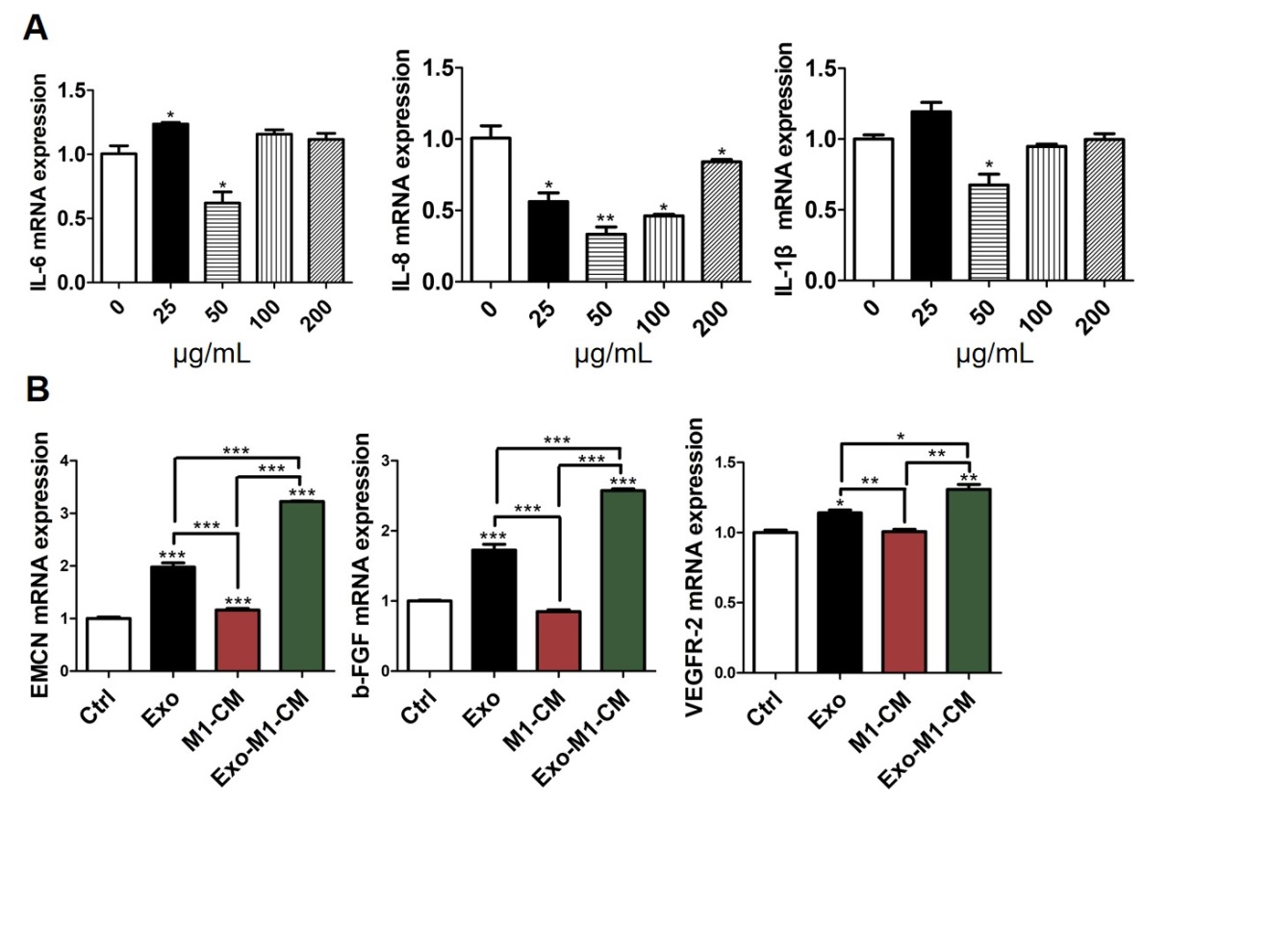


**Supplementary Figure 1.** (A) Serum-Exo concentration optimization for macrophage inflammation inhibition in vitro by RT-qPCR analysis. (B) The direct effect of serum-Exo on angiogenic differentiation markers’ expression in HUVEC cells. The significant difference between the groups, **p<0.05, **p<0.01, and ***p<0.001*.


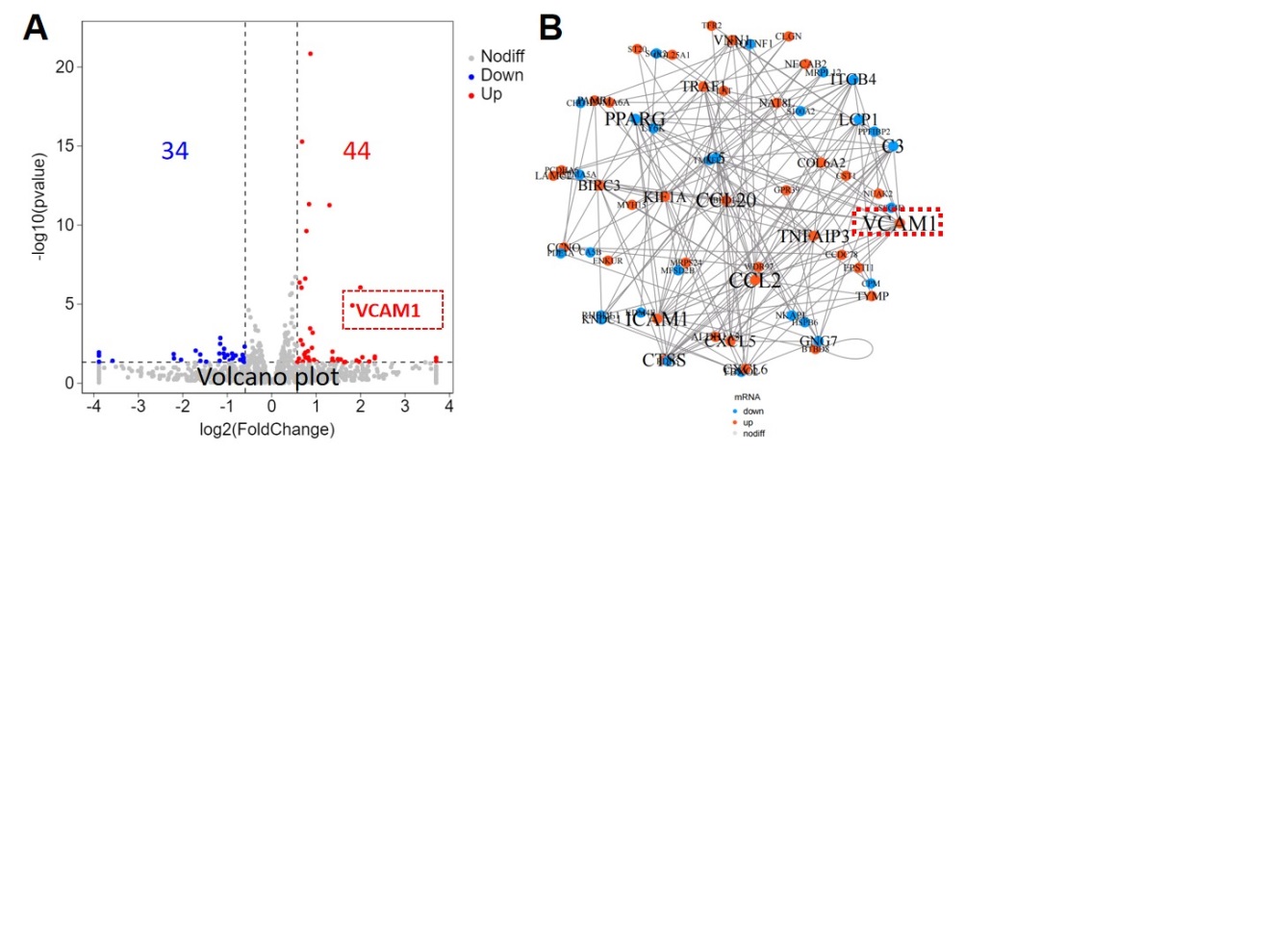


**Supplementary Figure 2.** (A) The volcano plot of differentially expressed genes in HUVEC cells treated with serum-Exosome-treated M1 macrophage-CM and M1 macrophage-CM. (B) Protein-Protein Interaction Networks (PPI) show the interaction of upregulated (red) and downregulated (blue) genes during the angiogenic differentiation of HUVEC cultured with serum-Exosome-treated M1 macrophage-CM treatment. (1.5-fold with p < 0.05)
